# miRNA-dependent target regulation: functional characterization of single-nucleotide polymorphisms identified in genome-wide association studies of Alzheimer’s disease

**DOI:** 10.1186/s13195-016-0186-x

**Published:** 2016-05-24

**Authors:** Charlotte Delay, Benjamin Grenier-Boley, Philippe Amouyel, Julie Dumont, Jean-Charles Lambert

**Affiliations:** NSERM U1167, Facteurs de risque et déterminants moléculaires des maladies liées au vieillissement (RID-AGE) Research Group, Lille, France; Institut Pasteur de Lille, Lille, France; University of Lille, Lille, France

**Keywords:** MicroRNA, PolymiRTS, Single-nucleotide polymorphism, Alzheimer’s disease, FERMT2, NUP160

## Abstract

**Background:**

A growing body of evidence suggests that microRNAs (miRNAs) are involved in Alzheimer’s disease (AD) and that some disease-associated genetic variants are located within miRNA binding sites. In the present study, we sought to characterize functional polymorphisms in miRNA target sites within the loci defined in earlier genome-wide association studies (GWAS). The main objectives of this study were to (1) facilitate the identification of the gene or genes responsible for the GWAS signal within a locus of interest and (2) determine how functional polymorphisms might be involved in the AD process (e.g., by affecting miRNA-mediated variations in gene expression).

**Methods:**

Stringent in silico analyses were developed to select potential polymorphisms susceptible to impairment of miRNA-mediated repression, and subsequent functional assays were performed in HeLa and HEK293 cells.

**Results:**

Two polymorphisms were identified and further analyzed in vitro. The AD-associated rs7143400-T allele (located in 3′ untranslated region [3′-UTR] of *FERMT2*) cotransfected with miR-4504 resulted in lower protein levels relative to the rs7143400-G allele cotransfected with the same miRNA. The AD-associated rs9909-C allele in the 3′-UTR of *NUP160* abolished the miR-1185-1-3p-regulated expression observed for the rs9909-G allele.

**Conclusions:**

When considered in conjunction with the findings of previous association studies, our results suggest that decreased expression of *FERMT2* might be a risk factor in the etiopathology of AD, whereas increased expression of *NUP160* might protect against the disease. Our data therefore provide new insights into AD by highlighting two new proteins putatively involved in the disease process.

**Electronic supplementary material:**

The online version of this article (doi:10.1186/s13195-016-0186-x) contains supplementary material, which is available to authorized users.

## Background

Alzheimer’s disease (AD) is the most common form of dementia worldwide. Despite extensive efforts, knowledge of AD pathophysiology is still incomplete. Understanding the genetics of AD might be one of the best ways of improving knowledge of its underlying pathophysiological processes. Indeed, the estimated heritability for the common late-onset forms of AD is between 60 % and 80 % [1], suggesting that most of the pathophysiological pathways in AD are driven by (or at least influenced by) genetic determinants. Moreover, the emergence of genomic approaches (such as genome-wide association studies [GWASs]) has enabled the characterization of many different genetic loci associated with AD risk and have given support to the genetic hypothesis in AD [2–8]. However, a locus of interest may encompass dozens of genes; in many cases, the most functionally relevant gene (i.e., the one responsible for the GWAS signal) has not yet been identified. Indeed, functional variants have rarely been characterized (despite specific efforts in this respect), and potential functional variants have been characterized in only six GWAS-defined genes so far: *SORL1*, *BIN1*, *CR1*, *CLU*, *ABCA7*, and *CD33* [9]. It is noteworthy that none of the potential variants within these genes have yet been linked to modulations in microRNA (miRNA or miR) binding. However, there is a growing body of evidence to suggest that miRNAs are involved in AD and that disease-associated genetic variants may be located within miRNA binding sites. This has already been observed in several types of disease, including hypertension [10]; nephropathy [11]; cancer [12, 13]; and neurological diseases such as Tourette syndrome [14], schizophrenia [15], cerebral amyloid angiopathy, and AD [16–18]. We therefore decided to characterize functional polymorphisms in miRNA target sites (PolymiRTSs) located within the GWAS-defined loci and associated with the AD risk. Our particular objectives were to (1) facilitate the identification of the gene or genes responsible for the GWAS signal within a locus of interest and (2) determine how these polymorphisms are involved in the AD process (e.g., by affecting miRNA-mediated variations in gene expression).

miRNAs are small (approximately 21 nucleotides) RNAs that interact with the 3′ untranslated region (UTR) of their target mRNA transcripts by partial sequence complementarity (resulting in destabilization of the mRNA and/or inhibition of translation) [19]. Interfering with this function (either by altering existing miRNA binding sites or by creating new, illegitimate miRNA binding sites) may thus result in significant downstream effects on protein expression and disease phenotypes [20]. The function of miRNAs depends primarily on the miRNA seed region (nucleotides 2–8 of the mature sequence), which is the smallest region required for binding to the target mRNA [21, 22]. Although the seed region is the critical component in target recognition, other parameters (such as 3′ complementarity, Adenosine -Uracile density around the target site, and the location within the 3′-UTR sequence) influence the binding affinity [23, 24]. Various algorithms for miRNA target prediction have been developed (each with its own set of rules). In the present study, we used the TargetScan [24], miRANDA [25], and TargetSpy [26] algorithms to identify PolymiRTSs in the AD-associated loci identified in our previous GWAS [8].

## Methods

This study is based on the publicly available International Genomics of Alzheimer’s Project (IGAP) database and did not need specific ethical approval. All the necessary consents were independently obtained by each consortium in the IGAP database as specified elsewhere [8].

### Identification of miRNA target sites in AD-associated genes and data mining

**Fig. 1:**
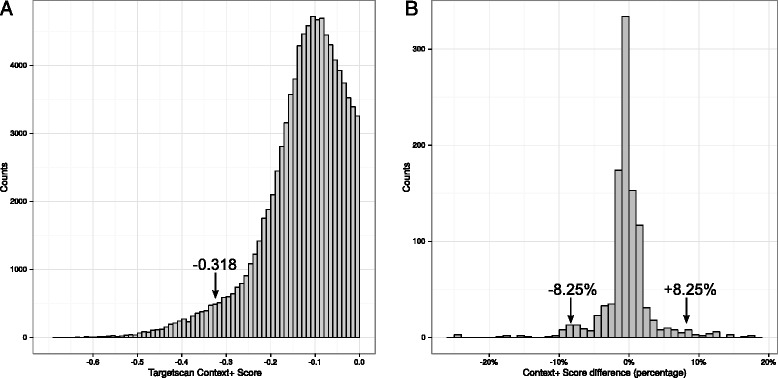
In silico approach for assessing score thresholds for identification of microRNA (miRNA) target sites and polymorphisms in a microRNA target site (PolymiRTSs). **a** A graphical representation of the “basic score filtering” in TargetScan. The “context +” scores for each miRNA binding site are plotted on the *x*-axis, and the number of counts with which each score was found is plotted on the *y*-axis. The top 5 % context + scores had a value below −0.318. **b** A graphical representation of the “score difference filter” in TargetScan. The percentage change in the context + scores for each miRNA binding site (due to the presence of a PolymiRTS) is plotted on the *x*-axis, and the number of counts with which each change in score was found is plotted on the *y*-axis. The top 5 % context + score changes had a value below −8.25 % or above 8.25

When considering the 220 genes in 29 AD-associated loci, we downloaded the reference 3′-UTR sequences from the UCSC Table Browser (using the human assembly GRCh37/Hg19) and loaded them into the miRANDA (version 3.3a), TargetScan (version 6.2), and TargetSpy (version 1) software [26–29]. TargetScan allows filtering based on cross-species target site conservation to focus on biologically relevant sites. However, given that AD is a pathology that manifests itself only in humans, we also chose to include less conserved (and perhaps more human-specific) miRNA sites [30]. To identify target sites in the reference sequences, we initially applied the following filters: a TargetScan context + score <0, a miRANDA prediction score >140 (which corresponds to a perfect seed match, with no other alignments), TargetSpy’s sensitive setting [26], and canonical sites only. The target sites predicted by miRANDA and TargetScan were then filtered (“basic score filter”) by ranking the scores for each algorithm independently and setting a threshold at 5 % of the “best scores” (a TargetScan context + score less than −0.318 and a miRANDA prediction score greater than 163). This approach selected the miRNA target sites with the best score and thus increased the likelihood of predicting biologically relevant sites. A graphic illustration of the application of this principle to TargetScan filtering is given in Fig. 1a. To identify target sites affected by single-nucleotide polymorphisms (SNPs), we generated in silico 3′-UTR sequences for each of the genes of interest, containing only the major alleles of all SNPs described in the 1000 Genomes database (with a frequency >1 % in the European ancestry panel, no deletions and insertions, 1000 Genomes Phase 1 integrated release version 3 haplotypes, 2010–11 data freeze, 14 Mar 2012 haplotypes) [31]. We then generated several 3′-UTR sequences, each containing the minor allele of one of these SNPs and the major alleles of all other SNPs. These sequences were then analyzed using the algorithms and filters described above. The corresponding sites in major or minor allele-bearing sequences were compared. The sites affected in their 3′ supplementary region were filtered, and only those with the greatest effects were retained. Therefore, we ranked these sites according to the percentage change in score when comparing major and minor allele sequences. We then filtered these sites (“score difference filter”) to keep only the top 5 % highest score differences; this corresponded to score differences below −8.25 % or above 8.25 %. The application of this principle to TargetScan filtering is shown in Fig. 1b. After these filtering steps (based on the predicted target sites’ scores and differences between scores), we selected sites predicted by at least two of three algorithms (“multiple algorithm filter”). These selected target sites were filtered for association between the PolymiRTS and AD (*p* value threshold 5 × 10^−6^) [8]. Next, we compared target sites on each allele, but without applying the basic score filter. We then selected target sites affected by the four SNPs identified in the first part of the analysis (rs7143400, rs610932, rs2847655, rs9909). Again, we selected target sites predicted by at least two algorithms. Last, target sites were filtered for alterations (as reported in the scientific literature) in the expression of the corresponding miRNA in AD.

### cDNA constructs and mutagenesis

The *MS4A6A* 3′-UTR was amplified from blood using the primers listed in Additional file 1: Table S1. Mutagenesis of the *NUP160*, *FERMT2* (Switchgear Genomics, Menlo Park, CA, USA), and *MS4A6A* 3′-UTRs was performed using the primers listed in Additional file 1: Table S1. The polymerase chain reaction products were inserted downstream of the luciferase gene in the psiCHECK2 vector (Promega, Madison, WI, USA). Mutagenesis of the *MS4A2* 3′-UTR (Switchgear Genomics) was performed using the QuikChange II Site-Directed Mutagenesis Kit (Agilent Technologies, Santa Clara, CA, USA). Both wild-type and mutated 3′-UTRs were cloned in the pLightSwitch vector (Switchgear Genomics).

### Cell culture and transfection

Human HeLa and HEK293 cells were respectively cultured in Eagle’s minimal essential medium (American Type Culture Collection, Teddington, UK) and DMEM/Ham’s F-12 1:1 medium (Life Technologies, Carlsbad, CA, USA) supplemented with 10 % heat-inactivated fetal bovine serum. One day before transfection, HeLa cells were plated at a density of 50,000 cells/cm^2^ and HEK293 cells were plated at 125,000 cells/cm^2^. Transfection was performed using Attractene reagent (QIAGEN, Venlo, The Netherlands) according to the manufacturer’s instructions.

### Luciferase reporter assays

Cells were transfected with 50 nM premiRs (QIAGEN) and 100 ng/cm^2^ psiCHECK2/3′-UTR (Promega) or pLightSwitch/3′-UTR plasmids (Switchgear Genomics). Twenty-four hours posttransfection, cells were lysed and luciferase activity was measured using the Dual-Glo® Luciferase Assay System (Promega) and a Wallac Victor luminometer (Promega) according to the manufacturer’s instructions. All experiments were performed at least three times in triplicate, and statistical analyses using the Mann-Whitney *U* test were performed with R software (version 3.2.2; https://www.R-project.org/).

## Results

### In silico identification of single-nucleotide polymorphisms with a possible functional impact on miRNA-mediated regulation of expression

**Fig. 2:**
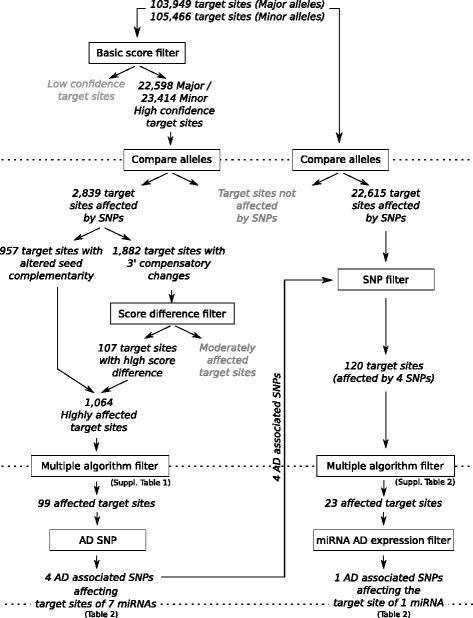
A graphical representation of the workflow used to identify microRNA (miRNA) target sites and polymorphisms in a microRNA target site. The 103,949 possible “major allele” target sites and the 105,466 possible “minor allele” target sites identified by TargetScan, miRANDA, and TargetSpy were filtered (using a “basic score filter”) to produce a list of high-confidence target sites. We then compared major and minor allele-bearing sequences to identify target sites affected by the presence of single-nucleotide polymorphisms (SNPs). Target sites affected in the 3′ supplementary region were subjected to the “score difference filter.” For all sites affected within the seed region and those having passed the “score difference filter,” we compared the results produced by TargetScan, miRANDA, and TargetSpy. Only sites predicted by at least two of the algorithms (the “multiple algorithm filter”) were selected. The selected sites were filtered on the basis of association between the SNP and Alzheimer’s disease (AD). We next assessed other miRNA target sites possibly affected by the four identified SNPs (no basic score filtering + SNP filter). We again applied the multiple algorithm filter. Only one of these miRNAs was also known to be deregulated in the AD brain (the “miRNA AD expression filter”)

We first generated an artificial 3′-UTR sequence in silico containing the major alleles of all known SNPs within the 220 genes in AD-associated loci [8]. On this basis, we generated sequences containing each of the minor alleles in turn. This enabled us to define all the potential miRNA binding sites within the 3′-UTR sequences (including those created by the presence of the minor SNP alleles). By using three stand-alone algorithms (TargetScan, TargetSpy, and miRANDA), we predicted 103,949 possible miRNA binding sites for the “major allele” sequence and 105,466 for the “minor allele” sequences (Fig. 2). We next used a “basic score filter” (see the Methods section) to select only high-confidence target sites. This step isolated 22,598 “major allele” sites and 23,414 “minor allele” sites (Fig. 2) for further analysis.

We then compared the major and minor allele sequences to establish whether SNPs within the 3′-UTRs might affect miRNA binding. A total of 2839 binding sites were predicted (by at least one of the three algorithms) to be modified by the presence of SNPs. The modifications associated with these SNPs were divided into two categories: (1) those that disrupted or created new canonical sites (i.e., sites with perfect complementarity to the miRNA seed sequence [*n* = 957]) (Fig. 2) and (2) those that affected the binding affinity without changing the target site’s complementarity to the miRNA seed region (target site complementarity is affected in the 3′ supplementary region [*n* = 1882] [24]). Although SNPs that alter canonical complementarity are expected to have strong effects on miRNA binding, the effects of SNPs that modify the prediction score (but not the seed complementarity) are more difficult to assess. We therefore used another strict “score difference filter” (see the Methods section) to select SNPs with the largest effects on miRNA binding. This reduced the number of sites affected in the 3′ supplementary region from 1882 to 107 (Fig. 2). By pooling these latter sites (*n* = 107) with the disrupted and/or new canonical sites (*n* = 957), we retained a total of 1064 sites of interest. Ninety-nine of these possible PolymiRTSs were simultaneously identified by two or more algorithms (the “multiple algorithm filter”); of those 99 sites, 37 were created, 51 were disrupted, 10 changed their site type, and only 1 site was affected in the 3′ supplementary region upon introduction of the minor allele (Fig. 2 and Additional file 2: Table S2).

Although all 99 miRNA binding sites might be of biological interest, we further narrowed down this list by focusing on SNPs with relevance for AD risk (i.e., selected by GWAS with a *p* value below 5 × 10^−6^) [8]. With this approach, we identified four AD-associated SNPs in seven miRNA target sites (miR-4504, miR-626, miR-6876-3p, miR-6888-3p, miR-3945, miR-585-3p, miR-3976). These potentially AD-associated PolymiRTSs were located within the 3′-UTR of the *MS4A*2, *MS4A6A*, *NUP160*, and *FERMT2* genes (Table 1).

**Table 1:** Alzheimer’s disease-associated polymorphisms in a microRNA target sites identified in silico, with the corresponding minor allele frequency, odds ratio, and potential effect on microRNA binding

Gene	PolymiRTS	Minor allele	MAF	OR	95 % CI	miRNA	PolymiRTS consequence	Anticipated effect
*FERMT2*	rs7143400	T	10.08 %	1.09	1.04–1.15	hsa-miR-4504	Creation perfect seed	Decreased expression
*MS4A2*	rs2847655	C	41.09 %	0.90	0.87–0.93	hsa-miR-585-3p	Disruption perfect seed	Increased expression
						hsa-miR-3945	Creation perfect seed	Decreased expression
						hsa-miR-6876-3p	Disruption perfect seed	Increased expression
*MS4A6A*	rs610932	A	42.49 %	0.91	0.88–0.94	hsa-miR-626	Disruption perfect seed	Increased expression
						hsa-miR-6888-3p	Creation perfect seed	Decreased expression
*NUP160*	rs9909	C	33.75 %	0.93	0.90–0.96	hsa-miR-3976	Creation perfect seed	Decreased expression
						hsa-miR-1185-1-3p	Disruption perfect seed	Increased expression

*MAF* minor allele frequency, *PolymiRTS* polymorphism in a microRNA target site, *miR* and *miRNA* microRNA

A summary of the genes, single-nucleotide polymorphisms, minor allele identity relative to 3′ untranslated region strand, MAF, and OR [95 % CI] (in the International Genomics of Alzheimer’s Project database discovery or meta-analysis study when available [8]), affected miRNAs, the effects of the Alzheimer’s disease-associated PolymiRTSs identified in this study, and the predicted consequences

We next looked at whether these four SNPs could affect other relevant miRNA binding sites. In fact, our use of stringent filters to characterize the potential impact of PolymiRTSs of interest may have excluded some relevant results. We therefore determined which of the 103,949 “major allele” target sites and 105,466 “minor allele” target sites might be altered by these 4 SNPs in the absence of score filters. Of the initial 22,615 target sites affected by any SNP, 120 were affected by any 1 of the 4 AD-associated SNPs being studied. These included drastic effects such as disruption or creation of sites as well as all slight alterations in binding score between the 3′-UTRs and their miRNAs that might be binding to their targets some dozen base pairs away from the SNPs being studied. Application of the above-mentioned “multiple algorithm filter” (i.e., sites predicted by at least two algorithms) selected 23 miRNAs, including the 7 miRNAs described above (Fig. 2 and Additional file 3: Table S3). An in-depth analysis of the literature revealed that only 5 of the 23 miRNAs had been studied previously in the context of AD. miRNA-206, miR-1, and miR-654-5p were found not to be deregulated in AD tissue versus control tissue [32–35], whereas the results for miR-30a-3p varied between studies [32, 34–37]. Importantly, researchers in two independent studies had found that levels of miR-1185-1-3p were abnormally low in the AD brain (compared with controls) [35, 38] (Additional file 3: Table S3). Hence, only miR-1185-1-3p was added to our initial list of seven miRNAs (Table 1).

In summary, we identified four PolymiRTSs likely to modulate the impact of eight different miRNAs (Fig. 2).

### Assessment of the biological function of the identified miRNA target sites

**Fig. 3:**
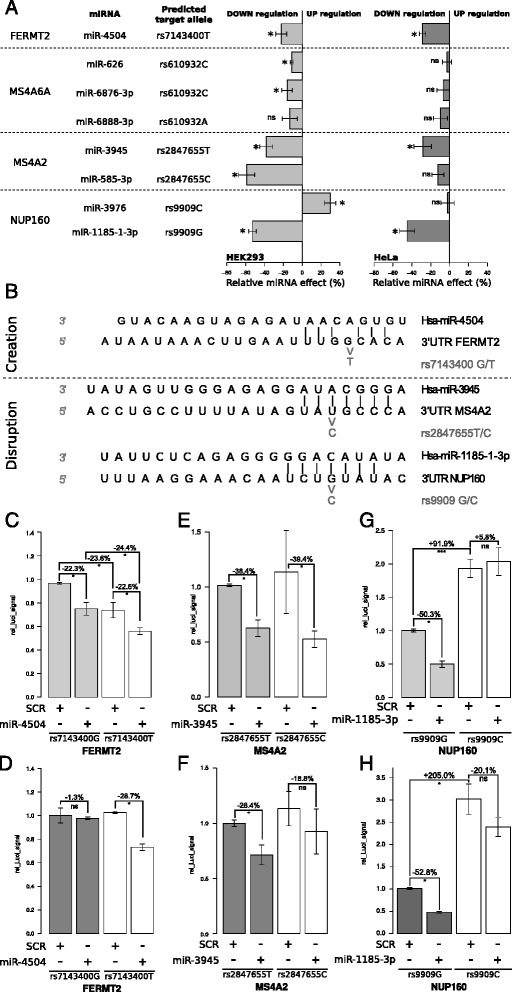
Identification of functional microRNA (miRNA, miR) target sites and polymorphisms in a microRNA target site (PolymiRTSs) in HEK293 and HeLa cells. **a** Assessment of the relative effect of miRNAs on their predicted targets using luciferase reporter assays in HEK293 and HeLa cells. Luciferase constructs bearing the 3′ untranslated region (3′-UTR) of *FERMT2*, *MS4A2*, *MS4A6A*, and *NUP160* were cotransfected with miR-4504, miR-585-3p, miR-3945, miR-626, miR-6867-3p, miR-6888-3p, miR-3976, miR-1185-1-3p, or scrambled (SCR) control miRNA. Each miRNA was cotransfected with the best predicted target allele, as indicated. Changes in lysate luciferase activity of the miRNA-transfected cells (relative to SCR control miRNA transfected cells) are shown. Negative and positive values indicate decreased and increased expression, respectively, compared with an SCR control miRNA. **b** Alignment between miR-4504, miR-3945, and miR1185-1-3p and 3′-UTRs of *FERMT2*, *MS4A2*, and *NUP160*. The physical consequences (creation and/or disruption of perfect seed matches) of minor allele PolymiRTS are indicated. (**c** and **d**) Luciferase assays showing the effect of rs7143400-G/T on the repressor activity of miR-4504 with regard to the 3′-UTR of *FERMT2* in (**c**) HEK293 cells and (**d**) HeLa cells. (**e** and **f**) Luciferase assays showing the effect of rs2847655-T/C on the repressor activity of miR-3945 with regard to *MS4A2* in (**e**) HEK293 cells and (**f**) HeLa cells. (**g**) and (**h**) Luciferase assays showing the effect of rs9909G/C on the repressor activity of miR1185-1-3p with regard to *NUP160* in (**g**) HEK293 cells and (**h**) HeLa cells. *p < 0.05 by Mann-Whitney *U* test; ***p < 0.001 by Mann-Whitney *U* test; *ns* not significant by Mann-Whitney *U* test. The quoted data correspond to the average of the mean of at least three independent experiments performed in triplicate. The standard error of the mean is indicated on the graphs

We first used luciferase activity assays in two independent cell-based models (HeLa and HEK293 cell lines) to assess the function of the eight miRNA target sites found within the respective 3′-UTRs of the *FERMT2*, *MS4A6A*, *MS4A2*, and *NUP160* genes. We observed that six of the eight putative target sequences were associated with downregulation of the luciferase activity in HEK293 cells when cotransfected with their targeting miRNAs (relative to control miRNAs), whereas three were associated with downregulation in HeLa cells (Fig. 3a).

The luciferase activities of HEK293 and HeLa cells transfected with a *FERMT2*:rs7143400-T 3′-UTR luciferase construct were, respectively, 22.3 ± 5.8 % and 28.7 ± 3.3 % lower in the presence of miRNA-4504 (relative to a scrambled [SCR] control miRNA). The luciferase activities of the *MS4A6A*:rs610932-C 3′-UTR reporter constructs were, respectively, 11.4 ± 1.3 % and 16.2 ± 5.5 % lower than SCR controls when cotransfected with miR-626 or miR-6876-3p in HEK293 cells. This was not observed in HeLa cells. When the *MS4A6A*:rs610932-A luciferase constructs were cotransfected with miR-6888-3p, no significant differences in luciferase activity were observed in either cell line.

Compared with SCR control miRNAs, we observed downregulation by, respectively, 38.4 ± 6.7 % and 59.1 ± 8.6 % when miR-3945 or miR-585-3p was cotransfected with *MS4A2* 3′-UTR luciferase constructs in HEK293 cells. The downregulation observed with miR-3945 was also observed in HeLa cells (by 28.4 ± 9.3 %), whereas cotransfection with miR-585-3p did not significantly alter luciferase activity in this cell line.

Cotransfection of *NUP160*:rs9909-C 3′-UTR luciferase constructs with miR-3976 did not modify the luciferase activity in HeLa cells but increased it in HEK293 cells (29.3 ± 5.9 %). Last, when miR-1185-1-3p was cotransfected with *NUP160*:rs9909-G 3′-UTR luciferase constructs, decreases of 52.8 ± 4.1 % and 45.0 ± 8 % in luciferase activity were observed in HEK293 and HeLa cells, respectively (compared with SCR control miRNAs).

In summary, *FERMT2*:rs71433400-T, *MS4A2*:rs2847655-T, and *NUP160*:rs9909-G transcript alleles were downregulated in both HEK293 and HeLa cells by miR-4504, miR-3945, and miR1185-1-3-p, respectively. *MS4A6A*:rs610932-C and *MS4A2*:rs2847655-C were respectively targeted by miR-626/miR-6876-3p and miR-585-3p, but this occurred in a cell-dependent manner. Although the latter miRNAs may be of some interest, we chose to focus on miRNAs with marked effects in both cell lines.

### Biological validation of the PolymiRTSs identified in silico

We next looked at whether allelic variations in miRNA binding sequences might affect the impact of the miRNAs being studied on target regulation. As seen from the sequence alignment of *FERMT2* 3′-UTR and miR-4504, the minor rs7143400-T allele creates an illegitimate canonical binding site compared with the major rs7143400-G allele (Fig. 3b). The presence of rs7143400-G is thus expected to decrease miR-4504’s downregulating effect on the *FERMT2* 3′-UTR reporter levels (Fig. 3a). However, the differences in luciferase activity for HEK293 cells cotransfected with miR-4504 (relative to SCR control miRNA) were similar for rs7143400-T- and rs7143400-G-bearing constructs (i.e., decreases of 22.6 ± 7.1 % and 22.3 ± 5.8 %, respectively) (Fig. 3c). Nevertheless, we observed decreases in luciferase activity of 23.6 ± 6.6 % and 24.4 ± 7.5 % between rs7143400-G and rs7143400-T when coexpressed with either SCR control miRNA or miR-4504, indicating an allelic effect independent of miRNA expression. In HeLa cells, this allelic difference was not found. However, miR-4504 decreased the expression of the reporter gene carrying the rs7143400-T allele (by 28.7 ± 3.3 %) but not that carrying the rs7143400-G allele (as expected on the basis of our in silico analysis) (Fig. 3d). In summary, and although the observations in HeLa and HEK293 cells were not identical, our data suggest that the rs7143400-T (minor) allele triggers *FERMT2* downregulation (compared with the rs7143400-G [major] allele) and that this downregulation might partially be linked to the presence of miR-4504.

As shown in Fig. 3b, the canonical binding site for miR-3945 on *MS4A2* corresponds to the major rs2847655-T allele, whereas the minor rs2847655-C allele disrupts perfect complementarity. As expected, cotransfection of HEK293 and HeLa cells with the *MS4A2*:rs2847655-T 3′-UTR luciferase constructs and miR-3945 resulted in a decrease in luciferase activity (by 38.4 ± 6.7 % and 28.4 ± 9.3 %, respectively, compared with SCR control miRNAs) (Fig. 3e, f). However, when miR-3945 was coexpressed with *MS4A2*:rs2847655-C 3′-UTR luciferase constructs, similar decreases in luciferase activity in *MS4A2*:rs2847655-T-expressing cells were observed in HEK293 and HeLa cells (39.4 ± 23.5 % and 18.8 ± 8.9 %, respectively) (Fig. 3e, f). Hence, miR-3945 appears to regulate *MS4A2* expression, but its function is probably not affected by rs2847655.

For *NUP160*:miR-1185-1-3p, the canonical binding site corresponds to the major rs9909-G allele, whereas the minor rs9909-C allele disrupts perfect complementarily. Accordingly, we expected that the minor allele would limit or abolish the effects of miR-1185-3p on the expression of *NUP160* 3′-UTR luciferase constructs. This prediction was confirmed in both cell lines. In fact, the rs9909-G allele was associated with significant decreases in luciferase activity in HEK293 and HeLa cells cotransfected with miR-1185-3p (by 52.8 ± 4.1 % and 52.6 ± 2.8 %, respectively, compared with SCR control miRNAs). In contrast, the rs9909-C allele completely disrupted this regulation in HEK293 and HeLa cells cotransfected with miR-1185-3p compared with SCR control miRNAs (Fig. 3g, h). It is noteworthy that the luciferase activity was significantly greater in HEK293 cells (by 91.9 ± 13.0 %) and HeLa cells (by 205 ± 31.1 %) cotransfected with the rs9909-C luciferase constructs and SCR control miRNAs compared with the rs9909-G allele cotransfected with the same miRNAs.

In summary, our data indicate that (1) compared with the rs9909-G allele, the rs9909-C allele may increase *NUP160* expression; and (2) at least part of this increase may be due to the disruption of a miR-1185-3p binding site.

## Discussion

The present study was focused on identifying functional SNPs located within AD-linked loci [8] that are likely to modulate miRNA binding. To predict the putative effects of AD-associated SNPs, we used the following criteria to select miRNA target site identification algorithms: (1) availability as a stand-alone program, (2) ability to query the latest version of the miRBase database, and (3) ability to handle an independent set of 3′-UTR sequences. The “gold standard” TargetScan, the less stringent miRANDA, and the machine learning algorithm TargetSpy met these criteria [21, 26, 27]. We could also have used algorithms (such as miRTarBase and miRecords) that take account of the miRNAs’ reported functionality by consulting collections of miRNA target data from both low- and high-throughput experiments [39, 40]. Although this approach would have increased the likelihood of finding legitimate sites, it would also have ruled out associations that have yet to be discovered. We therefore decided not to include these algorithms in our analysis.

We next defined a stringent approach for identifying miRNAs and PolymiRTSs with TargetScan, miRANDA, and TargetSpy, based on the alignment scores for miRNAs and their targets. In line with the thresholds used in other studies, we filtered our results with a TargetScan “context +” score of −0.318 [40–42]. In contrast, the miRANDA alignment scores used in the present study were far stricter than those reported in the literature [25, 27, 43]. We confirmed that three of the eight selected miRNA sites had a functional impact in two unrelated cell lines. Although we are aware that our approach may have excluded potentially interesting miRNAs and PolymiRTSs, we chose to focus on genomic sites and miRNAs that had functional importance in both cell lines [44]: the miRNAs miR-4504, miR-3945, and miR-1185-3p and the corresponding sites in the 3′-UTRs of the *FERMT2*, *MS4A2*, and *NUP160* genes. Our data showed that rs2847655 did not affect the decrease in luciferase activity observed when *MS4A2* 3′-UTR reporter constructs were coexpressed with miR-3945 (relative to coexpression with SCR control miRNAs). Furthermore, the AD-associated rs7043400-T allele was associated with lower *FERMT2* 3′-UTR luciferase reporter levels in HeLa cells in response to miR-4504 (compared with SCR control miRNAs). In HEK293 cells, the rs7043400-T allele was similarly associated with miR-4504-mediated repression, even though the rs7043400-G allele was also found to be regulated by miR-4504. Although the results in the two cell lines differed slightly, the presence of the rs7043400-T allele was always associated with decreased expression of the *FERMT2* 3′-UTR-dependent luciferase activity following miR-4504 overexpression. Since the rs7143400-T allele is associated with an increase in AD risk (OR 1.09, 95 % CI 1.04–1.15), our data suggest that low *FERMT2* expression might contribute to the development of AD. This hypothesis is in line with the results of recent screening experiments in *Drosophila*, where the authors identified the FERMT2 orthologs Fit1 and Fit2 as regulators of Tau toxicity; low expression of Fit1 or Fit2 exacerbated Tau toxicity in the *Drosophila* eye, whereas elevated expression resulted in the opposite phenotype [45]. Following an extensive analysis of the literature, we noted that many miRNAs predicted to target the FERMT2 mRNA are reportedly downregulated in AD (Table 2). Although the spatiotemporal expression pattern of FERMT2 in the brain and its regulating miRNAs have not yet been determined, this observation supports a hypothesis whereby dysregulation of miRNA expression and/or binding (due to polymorphisms such as rs7043400) may favor *FERMT2* underexpression and thus Tau pathology.

**Table 2:** Alzheimer’s disease-associated deregulation of microRNAs targeting *FERMT2* and *NUP160*

Gene	miRNA	AD	References
*FERMT2*	hsa-miR-29b-3p	Downregulated	Cogswell et al. [32], Hebert et al. [33], Nunez-Iglesias et al. [48], Geekiyanage et al. [49], Hebert et al. [35], Kiko et al. [50], Leidinger et al. [37], Tan et al. [51], Villa et al. [52], Denk et al. [34]
	hsa-miR-107	Downregulated	Hebert et al. [33], Wang et al. [53], Leidinger et al. [37], Muller et al. [54]
	hsa-miR-15a-5p	Downregulated	Cogswell et al. [32], Hebert et al. [33], Nunez-Iglesias et al. [46], Leidinger et al. [37], Denk et al. [34]
	hsa-miR-144-5p	Downregulated	Leidinger et al. [37], Denk et al. [34]
	hsa-miR-103a-3p	Downregulated	Cogswell et al. [32], Hebert et al. [33], Hebert et al. [35], Leidinger et al. [37], Denk et al. [34]
	hsa-miR-582-3p	Downregulated	Hebert et al. [35]
	hsa-miR-498	Not Altered	Hebert et al. [33]
	hsa-miR-29a-5p	Not Altered	Hebert et al. [35], Denk et al. [34]
	hsa-miR-222-3p	Not Altered	Hebert et al. [33], Lau et al. [38], Denk et al. [34]
	hsa-miR-424-5p	Upregulated	Cogswell et al. [32], Hebert et al. [33], Lau et al. [38], Denk et al. [34]
	hsa-miR-3163	Upregulated	Denk et al. [34]
*NUP160*	hsa-miR-1185-1-3p	Downregulated	Lau et al. [38]
	hsa-miR-126-5p	Downregulated	Cogswell et al. [32], Hebert et al. [35], Leidinger et al. [37], Denk et al. [34]
	hsa-miR-133b	Downregulated	Cogswell et al. [32], Hebert et al. [33], Denk et al. [34]
	hsa-miR-323b-3p	Upregulated	Leidinger et al. [37]

A summary of the miRNAs predicted to target *FERMT2* and *NUP160*, for which alterations in expression have been reported in Alzheimer’s disease (AD; mainly downregulated, upregulated, or not altered; refer to the quoted references). MicroRNAs (miRNA, miR) for which the literature results are ambiguous are not mentioned in the table

Last, we identified rs9909-C. This allele (1) is known to reduce AD risk (OR 0.93, 95 % CI 0.90–0.96) and (2) affected miR-1185-3p downregulation of the *NUP160* 3′-UTR luciferase construct. Our data indicate that increased NUP160 levels might be protective against the development of AD. The *NUP160* gene is located within the *CELF1* locus, and NUP160 is part of a protein family involved in nuclear transport. Interestingly, alterations in nuclear transport have been described as a possible mechanism in the pathogenesis of neurodegenerative diseases [46, 47]. Through extensive analysis of the literature, we noted that many miRNAs targeting the NUP160 mRNA (including miR-1185-3p) are reportedly downregulated in AD (Table 2) [35, 38], which might reflect an attempt of neurons to restore normal nuclear transport. Accordingly, it will be important to determine whether the *NUP160* gene accounts for the GWAS signal observed in the *CELF1* locus.

## Conclusions

We sought to identify functionally relevant genes in AD-associated loci by assessing the effect of AD-associated SNPs on the repressor activity of miRNAs. We identified rs7143400-T as being associated with low expression of the FERMT2 reporter (partially through regulation of miR-4504). We also validated the PolymiRTS rs9909-C in the 3′-UTR of *NUP160*, which was associated with elevated expression of the reporter as a result of miR1185-3p dysregulation. Last, our results suggest that (1) low expression of *FERMT2* might be an AD risk factor and (2) elevated expression of *NUP160* might protect against AD.

## Additional files

Additional file 1: Table S1. Primers used for cloning and mutagenesis of the 3′-UTRs of *FERMT2*, *MS4A2*, *MS4A6A* and *NUP160*. A summary of the base composition of the primers used for cloning and mutagenesis of the indicated gene 3′-UTRs. The mutated sites are the underlined bases. The purpose and direction of each primer are given. (XLS 21 kb) Additional file 2: Table S2. PolymiRTSs and the corresponding target genes and miRNAs identified in silico. A summary of the genes, PolymiRTSs, SNP major and minor alleles relative to 3′-UTR strand and their MAF (reported in the EUR panel of the 1000 Genomes database), the targeting miRNAs, and the predicted consequences. (XLS 33 kb) Additional file 3: Table S3. Other miRNA targeting sites identified by less stringent analysis near rs7143400-C/G, rs2847655-T/C, rs610923-C/A and rs9909-G/C. A summary of the genes, PolymiRTSs, effects of minor alleles, targeting miRNAs and miRNA expression alterations observed in AD (when available; refer to the cited references). The grayed miRNAs were also found in the stringent screening described in Fig. 2a in the main text. (XLS 23 kb)

## Abbreviations

AD: Alzheimer’s disease
GWAS: genome-wide association study
IGAP: International Genomics of Alzheimer’s Project
MAF: minor allele frequency
miR and miRNA: microRNA
PolymiRTS: polymorphism in a microRNA target site
SCR: scrambled
SNP: single-nucleotide polymorphism
UTR: untranslated region

## References

[1] Gatz M, Reynolds CA, Fratiglioni L, Johansson B, Mortimer JA, Berg S (2006). Role of genes and environments for explaining Alzheimer disease. Arch Gen Psychiatry..

[2] Harold D, Abraham R, Hollingworth P, Sims R, Gerrish A, Hamshere ML (2009). Genome-wide association study identifies variants at *CLU* and *PICALM* associated with Alzheimer’s disease. Nat Genet..

[3] Hollingworth P, Harold D, Sims R, Gerrish A, Lambert JC, Carrasquillo MM (2011). Common variants at *ABCA7*, *MS4A6A*/*MS4A4E*, *EPHA1*, *CD33* and *CD2AP* are associated with Alzheimer’s disease. Nat Genet..

[4] Lambert JC, Heath S, Even G, Campion D, Sleegers K, Hiltunen M (2009). Genome-wide association study identifies variants at *CLU* and *CR1* associated with Alzheimer’s disease. Nat Genet..

[5] Lambert JC, Grenier-Boley B, Harold D, Zelenika D, Chouraki V, Kamatani Y (2013). Genome-wide haplotype association study identifies the *FRMD4A* gene as a risk locus for Alzheimer’s disease. Mol Psychiatry..

[6] Naj AC, Jun G, Beecham GW, Wang LS, Vardarajan BN, Buros J (2011). Common variants at *MS4A4*/*MS4A6E*, *CD2AP*, *CD33* and *EPHA1* are associated with late-onset Alzheimer’s disease. Nat Genet..

[7] Seshadri S, Fitzpatrick AL, Ikram MA, DeStefano AL, Gudnason V, Boada M (2010). Genome-wide analysis of genetic loci associated with Alzheimer disease. JAMA..

[8] Lambert JC, Ibrahim-Verbaas CA, Harold D, Naj AC, Sims R, Bellenguez C (2013). Meta-analysis of 74,046 individuals identifies 11 new susceptibility loci for Alzheimer’s disease. Nat Genet..

[9] Van Cauwenberghe C, Van Broeckhoven C, Sleegers K. The genetic landscape of Alzheimer disease: clinical implications and perspectives. Genet Med. doi: 10.1038/gim.2015.117. 10.1038/gim.2015.117 PMC4857183 26312828

[10] Wei Z, Biswas N, Wang L, Courel M, Zhang K, Soler-Jover A (2011). A common genetic variant in the 3′-UTR of vacuolar H^+^-ATPase *ATP6V0A1* creates a micro-RNA motif to alter chromogranin A processing and hypertension risk. Circ Cardiovasc Genet..

[11] Papagregoriou G, Erguler K, Dweep H, Voskarides K, Koupepidou P, Athanasiou Y (2012). A miR-1207-5p binding site polymorphism abolishes regulation of *HBEGF* and is associated with disease severity in CFHR5 nephropathy. PLoS One..

[12] Bao BY, Pao JB, Huang CN, Pu YS, Chang TY, Lan YH (2011). Polymorphisms inside microRNAs and microRNA target sites predict clinical outcomes in prostate cancer patients receiving androgen-deprivation therapy. Clin Cancer Res..

[13] Nicoloso MS, Sun H, Spizzo R, Kim H, Wickramasinghe P, Shimizu M (2010). Single-nucleotide polymorphisms inside microRNA target sites influence tumor susceptibility. Cancer Res..

[14] Abelson JF, Kwan KY, O’Roak BJ, Baek DY, Stillman AA, Morgan TM (2005). Sequence variants in *SLITRK1* are associated with Tourette’s syndrome. Science..

[15] Gong Y, Wu CN, Xu J, Feng G, Xing QH, Fu W (2013). Polymorphisms in microRNA target sites influence susceptibility to schizophrenia by altering the binding of miRNAs to their targets. Eur Neuropsychopharmacol..

[16] Delay C, Calon F, Mathews P, Hébert SS (2011). Alzheimer-specific variants in the 3′UTR of amyloid precursor protein affect microRNA function. Mol Neurodegener..

[17] Delay C, Dorval V, Fok A, Grenier-Boley B, Lambert JC, Hsiung GY (2014). MicroRNAs targeting Nicastrin regulate Aβ production and are affected by target site polymorphisms. Front Mol Neurosci..

[18] Nicolas G, Wallon D, Goupil C, Richard AC, Pottier C, Dorval V (2016). Mutation in the 3′ untranslated region of APP as a genetic determinant of cerebral amyloid angiopathy. Eur J Hum Genet..

[19] Ambros V (2004). The functions of animal microRNAs. Nature..

[20] Sethupathy P, Collins FS (2008). MicroRNA target site polymorphisms and human disease. Trends Genet..

[21] Lewis BP, Shih I, Jones-Rhoades MW, Bartel DP, Burge CB (2003). Prediction of mammalian microRNA targets. Cell..

[22] Brennecke J, Stark A, Russell RB, Cohen SM (2005). Principles of microRNA-target recognition. PLoS Biol..

[23] Grimson A, Farh KKH, Johnston WK, Garrett-Engele P, Lim LP, Bartel DP (2007). MicroRNA targeting specificity in mammals: determinants beyond seed pairing. Mol Cell..

[24] Bartel DP (2009). MicroRNAs: target recognition and regulatory functions. Cell..

[25] Betel D, Koppal A, Agius P, Sander C, Leslie C (2010). Comprehensive modeling of microRNA targets predicts functional non-conserved and non-canonical sites. Genome Biol..

[26] Sturm M, Hackenberg M, Langenberger D, Frishman D (2010). TargetSpy: a supervised machine learning approach for microRNA target prediction. BMC Bioinformatics..

[27] Enright AJ, John B, Gaul U, Tuschl T, Sander C, Marks DS (2003). MicroRNA targets in *Drosophila*. Genome Biol..

[28] Karolchik D, Hinrichs AS, Furey TS, Roskin KM, Sugnet CW, Haussler D (2004). The UCSC Table Browser data retrieval tool. Nucleic Acids Res.

[29] Lewis BP, Burge CB, Bartel DP (2005). Conserved seed pairing, often flanked by adenosines, indicates that thousands of human genes are microRNA targets. Cell..

[30] Hu HY, He L, Fominykh K, Yan Z, Guo S, Zhang X (2012). Evolution of the human-specific microRNA miR-941. Nat Commun..

[31] Abecasis GR, Auton A, Brooks LD, DePristo MA, Durbin RM, Handsaker RE (2012). An integrated map of genetic variation from 1,092 human genomes. Nature..

[32] Cogswell JP, Ward J, Taylor IA, Waters M, Shi Y, Cannon B (2008). Identification of miRNA changes in Alzheimer’s disease brain and CSF yields putative biomarkers and insights into disease pathways. J Alzheimers Dis..

[33] Hébert SS, Horré K, Nicolaï L, Papadopoulou AS, Mandemakers W, Silahtaroglu AN (2008). Loss of microRNA cluster miR-29a/b-1 in sporadic Alzheimer’s disease correlates with increased BACE1/β-secretase expression. Proc Natl Acad Sci U S A..

[34] Denk J, Boelmans K, Siegismund C, Lassner D, Arlt S, Jahn H (2015). MicroRNA profiling of CSF reveals potential biomarkers to detect Alzheimer’s disease. PLoS One..

[35] Hébert SS, Wang WX, Zhu Q, Nelson PT (2013). A study of small RNAs from cerebral neocortex of pathology-verified Alzheimer’s disease, dementia with Lewy bodies, hippocampal sclerosis, frontotemporal lobar dementia, and non-demented human controls. J Alzheimers Dis..

[36] Hébert SS, Horré K, Nicolaï L, Bergmans B, Papadopoulou AS, Delacourte A (2009). MicroRNA regulation of Alzheimer’s amyloid precursor protein expression. Neurobiol Dis..

[37] Leidinger P, Backes C, Deutscher S, Schmitt K, Mueller SC, Frese K (2013). A blood based 12-miRNA signature of Alzheimer disease patients. Genome Biol..

[38] Lau P, Bossers K, Janky R, Salta E, Frigerio CS, Barbash S (2013). Alteration of the microRNA network during the progression of Alzheimer’s disease. EMBO Mol Med..

[39] Xiao F, Zuo Z, Cai G, Kang S, Gao X, Li T (2009). miRecords: an integrated resource for microRNA-target interactions. Nucleic Acids Res.

[40] Hsu SD, Lin FM, Wu WY, Liang C, Huang WC, Chan WL (2011). miRTarBase: a database curates experimentally validated microRNA-target interactions. Nucleic Acids Res.

[41] Jansen BJH, Sama IE, Eleveld-Trancikova D, van Hout-Kuijer MA, Jansen JH, Huynen MA (2011). MicroRNA genes preferentially expressed in dendritic cells contain sites for conserved transcription factor binding motifs in their promoters. BMC Genomics..

[42] Xiao Y, Xu C, Guan J, Ping Y, Fan H, Li Y (2012). Discovering dysfunction of multiple microRNAs cooperation in disease by a conserved microRNA co-expression network. PLoS One..

[43] John B, Enright AJ, Aravin A, Tuschl T, Sander C, Marks DS (2004). Human MicroRNA targets. PLoS Biol..

[44] Nam JW, Rissland OS, Koppstein D, Abreu-Goodger C, Jan CH, Agarwal V (2014). Global analyses of the effect of different cellular contexts on microRNA targeting. Mol Cell..

[45] Shulman JM, Imboywa S, Giagtzoglou N, Powers MP, Hu Y, Devenport D (2014). Functional screening in *Drosophila* identifies Alzheimer’s disease susceptibility genes and implicates Tau-mediated mechanisms. Hum Mol Genet..

[46] Sheffield LG, Miskiewicz HB, Tannenbaum LB, Mirra SS (2006). Nuclear pore complex proteins in Alzheimer disease. J Neuropathol Exp Neurol..

[47] Patel VP, Chu CT (2011). Nuclear transport, oxidative stress, and neurodegeneration. Int J Clin Exp Pathol..

[48] Nunez-Iglesias J, Liu CC, Morgan TE, Finch CE, Zhou XJ (2010). Joint genome-wide profiling of miRNA and mRNA expression in Alzheimer's disease cortex reveals altered miRNA regulation. PLoS One.

[49] Geekiyanage H, Jicha GA, Nelson PT, Chan C (2012). Blood serum miRNA: non-invasive biomarkers for Alzheimer's disease. Exp Neurol..

[50] Kiko T, Nakagawa K, Tsuduki T, Furukawa K, Arai H, Miyazawa T (2014). MicroRNAs in plasma and cerebrospinal fluid as potential markers for Alzheimer's disease. J Alzheimers Dis..

[51] Tan L, Yu JT, Hu N, Tan L (2013). Non-coding RNAs in Alzheimer's disease. Mol Neurobiol..

[52] Villa C, Ridolfi E, Fenoglio C, Ghezzi L, Vimercati R, Clerici F (2013). Expression of the transcription factor Sp1 and its regulatory hsa-miR 29b in peripheral blood mononuclear cells from patients with Alzheimer's disease. J Alzheimers Dis..

[53] Wang WX, Rajeev BW, Stromberg AJ (2008). Ren N, Tang G, Huang Q, al. The expression of microRNA miR- 107 decreases early in Alzheimer's disease and may accelerate disease progression through regulation of beta-site amyloid precursor protein-cleaving enzyme 1. J Neurosci.

[54] Müller M, Kuiperij HB, Claassen JA, Küsters B, Verbeek MM (2014). MicroRNAs in Alzheimer's disease: differential expression in hippocampus and cell-free cerebrospinal fluid. Neurobiol Aging..

